# Application of a Modified Generative Adversarial Network in the Superresolution Reconstruction of Ancient Murals

**DOI:** 10.1155/2020/6670976

**Published:** 2020-12-29

**Authors:** Jianfang Cao, Zibang Zhang, Aidi Zhao

**Affiliations:** ^1^School of Computer Science & Technology, Taiyuan University of Science and Technology, Taiyuan 030024, China; ^2^Department of Computer Science & Technology, Xinzhou Teachers University, Xinzhou 034000, China

## Abstract

Considering the problems of low resolution and rough details in existing mural images, this paper proposes a superresolution reconstruction algorithm for enhancing artistic mural images, thereby optimizing mural images. The algorithm takes a generative adversarial network (GAN) as the framework. First, a convolutional neural network (CNN) is used to extract image feature information, and then, the features are mapped to the high-resolution image space of the same size as the original image. Finally, the reconstructed high-resolution image is output to complete the design of the generative network. Then, a CNN with deep and residual modules is used for image feature extraction to determine whether the output of the generative network is an authentic, high-resolution mural image. In detail, the depth of the network increases, the residual module is introduced, the batch standardization of the network convolution layer is deleted, and the subpixel convolution is used to realize upsampling. Additionally, a combination of multiple loss functions and staged construction of the network model is adopted to further optimize the mural image. A mural dataset is set up by the current team. Compared with several existing image superresolution algorithms, the peak signal-to-noise ratio (PSNR) of the proposed algorithm increases by an average of 1.2–3.3 dB and the structural similarity (SSIM) increases by 0.04 = 0.13; it is also superior to other algorithms in terms of subjective scoring. The proposed method in this study is effective in the superresolution reconstruction of mural images, which contributes to the further optimization of ancient mural images.

## 1. Introduction

Ancient murals are the bright pearl in the treasure house of cultural heritage. At present, the protection of murals mostly focuses on the field research of ancient murals and the restoration of damaged areas of murals. For instance, Tong et al. [1] studied the paint layer and bottom layer of a Tang Dynasty tomb mural using spectral-domain optical coherence tomography. Wu et al. [2] proved the existence of casein in ancient Chinese mural pigments. Liang and Wan [3] proposed the idea of making color charts for Dunhuang frescoes to improve the color and spectral accuracy of digital imaging of cultural works of art. Cao et al. [4] proposed an ASB-LB algorithm to solve the problem of flake shedding of temple mural images. Sun et al. [5] proposed a line drawing generation method, which made mural images have different artistic styles. With the gradual development of ancient mural protection work and the maturity of superresolution reconstruction technology, mural image conservation will be further extended.

Image superresolution reconstruction refers to a technique that inputs one or more low-resolution images and outputs the corresponding high-resolution images through a specific algorithm. This will make mural images have a better effect on high-frequency detail information and overall image performance. From the perspective of algorithm types, superresolution reconstruction technology can be divided into interpolation-, reconstruction-, and learning-based superresolution reconstruction. Li and Orchard [6] proposed a new edge-oriented self-adaptive interpolation scheme. Based on the invariability of edge direction resolution, a high resolution was used to guide interpolations to enhance the resolution of still images. Interpolation-based methods have the advantages of low computational complexity and ease of understanding, but they also have some serious defects. Restored superresolution images often appear blurred or sawtooth. Li et al. [7] proposed a single image superresolution reconstruction method based on a genetic algorithm and regularization prior model. In this model, a genetic algorithm was used to search the solution space to avoid a local minimum value; then, the regularization prior model was used to perform a single point search in the solution space, and a higher quality superresolution reconstruction estimation was obtained. Zhao et al. [8] proposed a novel single image superresolution reconstruction method based on the unified partial differential equation, and the method achieved a good effect in enhancing the image edge and suppressing noise robustness. Zhang and He [9] proposed a single image superresolution reconstruction method based on mixed sparse representation, which was particularly effective for optimizing the reconstruction of noisy images. Bahy et al. [10] proposed a method based on local adaptive regularization parameters instead of fixed regularization parameters, which is convenient for addressing the reconstruction of low-resolution multifocus images. Nayak and Patra [11] proposed a new RSRR framework to keep the reconstructed image structure consistent. Dai et al. [12] proposed a method to represent soft edge smoothness based on SoftCuts measurements. This method first obtains the key information in the original image and combines the prior knowledge of the unknown superresolution image to constrain the generation of the corresponding superresolution image. Compared with the interpolation-based method, the above method has a better image superresolution reconstruction effect, but the method of constraining the generation of superresolution images by prior knowledge of unknown superresolution images may make the edge of superresolution images too sharp. Furthermore, the details of the image may become increasingly unstable with increasing image size.

In contrast, the method based on deep learning uses a large quantity of training data through multilayer nonlinear transformation to learn the corresponding relationship in some high-level abstract features between low-resolution images and high-resolution images and then realizes the superresolution reconstruction of the image according to the mapping relationship between the acquired images. Dong et al. [13] applied a convolutional neural network (CNN) to the field of superresolution reconstruction for the first time and proposed a deep-learning method for single image superresolution. For an input low-resolution image, the input image was first magnified to the target size by bicubic interpolation; then the nonlinear mapping between the interpolated low-resolution image and the high-resolution image was fitted by the CNN, and, finally, the reconstructed high-resolution image was output. However, this algorithm still retains part of the difference algorithm and does not thoroughly apply the idea of deep learning. Mao et al. [14] proposed a complete convolution encoding-decoding framework. This network consists of multiple convolution layers and deconvolution layers. Convolution layers capture the abstract content of the image and eliminate the damage; deconvolution layers upsample the features and restore image details; additionally, symmetric skips are introduced, which makes the training converge faster. However, this algorithm is likely to produce overfitting in superresolution reconstruction of mural image datasets. Huang et al. [15] proposed a multiframe superresolution method based on the consideration of image enhancement and image denoising. This method suppressed Gaussian noise and salt and pepper noise and made the edge of the reconstructed high-resolution image clear. However, the feature extraction ability of the algorithm is low, and, therefore, the superresolution reconstruction of the image with complex information is slightly fuzzy.

Anagun et al. [16] used a variety of loss functions to combine with the Adam optimizer for the selection of a satisfactorily convergent loss function. They also increased the residual module of the network to improve the performance of the model and used the Charbonnier or L1 loss function to reduce the time cost of model construction. Qin et al. [17] proposed a novel multiscale feature fusion residual network, which improved the expression ability of the network to obtain more accurate high-resolution images with satisfactory accuracy and visual effect. Zhang and An [18] introduced a superresolution reconstruction method based on migration learning and deep learning, which can not only obtain high-quality, high-resolution images but also reduce the time cost of model construction. Ledig et al. [19] proposed the SRGAN algorithm and designed a loss function to enhance the reality of the restored image. In their method, the adversarial loss function of the generative adversarial network (GAN) was combined, which enables the output superresolution image to be more authentic. Lim et al. [20] proposed the EDSR algorithm, which removed batch standardization, reduced the space used during training, and removed the unnecessary modules in the traditional residual network. To improve the efficiency of high-resolution image reconstruction, Mei et al. [21] extended traditional nonlocal attention to a new cross-scale nonlocal attention to model cross-scale self-similarity. Jiang et al. [22] proposed a hierarchical dense connection network structure to improve the efficiency of superresolution reconstruction. Yi et al. [23] proposed a multitemporal ultradense memory network for video superresolution, which expanded the width of the network and reduced the layer depth to reduce computational complexity. Jiang et al. [24] proposed a GAN-based edge-enhancement network, based on which clearer images were obtained, compared with previous GAN-based methods. All of the above learning-based superresolution reconstruction algorithms optimize the network structure and loss function from different perspectives and solve specific problems. However, due to the characteristics of mural images, such as small image datasets, uneven image quality, and rich image color, there are still many defects in the direct application of the existing superresolution reconstruction methods, such as fuzzy restoration of important texture information of the image, impure color in the reconstructed image, and changes in the overall artistic effect of the original image artistic.

Based on the aforementioned information, this study proposes a new superresolution reconstruction algorithm, which is applied to the superresolution reconstruction of ancient mural images. The improvement of the proposed algorithm is mainly as follows:

1. The network design takes GAN as the basic framework, including the generative network and the discriminate network; MSE loss, VGg loss, and adversarial loss functions are introduced to optimize the network in two stages.
2. The generative network is based on CNN, in which deconvolution operation is replaced by subpixel convolution, batch standardization is removed, and residual module is introduced to deepen the network to optimize the network structure.
3. The discriminant network increases the number of network layers, and residual modules are integrated to enable the network to extract more image information, and the expression ability of the discriminant network is increased to further optimize the generative network model.

## 2. Methodology

### 2.1. GAN

Since GAN was first proposed by Ian Goodfellow in 2014 [25], there has been a new upsurge of research. GAN is composed of generators and discriminators. The generator is responsible for generating samples, and the discriminator is responsible for determining whether the samples generated by the generator are true. The generator should confuse the discriminator as much as possible, and the discriminator should distinguish the samples generated by the generator from the real samples as much as possible.

**Figure 1:**
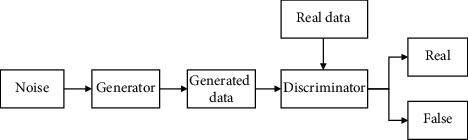
Basic structure of GAN [25].

The basic structure of the GAN is illustrated in Figure 1.

The target function of GAN is as follows:

(1) $$\begin{array}{c} \underset{G}{\min}\underset{D}{\max}V\left(D,G\right)=E_{x∼p_{\text{data}}\left(x\right)}\left[\log\;D\left(x\right)\right]+E_{z∼p_{z}\left(z\right)}\left[\log\left(1-D\left(G\left(z\right)\right)\right)\right]. \end{array}$$

In the first part, the optimization of the discriminator is realized through max_*D*_*V*(*D*, *G*), *V*(*D*, *G*) is the objective function of the discriminator, and the first item *V*(*D*, *G*) represents the mathematical expectation of the probability of the samples from the real data distribution, which are determined as the real samples by the discriminator. For the sample from the real data distribution, the closer the probability of being predicted as a positive sample is to 1, the better. The second item *E*_*z*∼*p*_*z*_(*z*)_[log(1 − *D*(*G*(*z*)))] represents the expectation for the negative logarithm of the prediction probability by the discriminator for the image generated by the generator that originates from the noise distribution *p*_*z*_(*z*). A higher expectation value indicates a better performance of the discriminator.

In the second part, the optimization of the generator is realized through min_*G*_max_*D*_*V*(*D*, *G*). The generator is not the objective function of the minimized discriminator min_*G*_*V*(*D*, *G*) but the maximum value of the objective function of the minimized discriminator. The maximum value of the objective function of the discriminator represents the Jensen-Shannon (JS) divergence between the distribution of the real data and that of the generated data. JS divergence can measure the similarity of distributions. The closer the two distributions are, the smaller the JS divergence will be.

### 2.2. Residual Network

**Figure 2:**
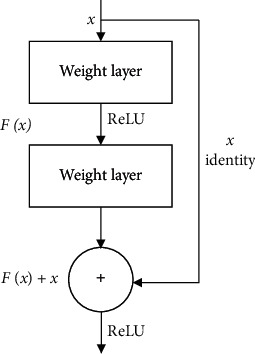
Basic structure of the residual module [26].

By increasing the number of network layers in the CNN, more abstract and semantic image features can be extracted. However, simply increasing the number of layers of the network causes gradient dispersion and degradation, which may eventually lead to saturation or even decline in the accuracy of the model in the training set. To solve this problem, He et al. [26] proposed a residual network (ResNet) and achieved satisfactory results in the classification task in the ImageNet competition. For its simple and practical characteristics, ResNet has been widely used in the fields of target detection, image segmentation, and text recognition. The basic structure of the residual module is shown in Figure 2.

In the figure, *X* represents the input of the residual block of the current layer, *F* (*x*) represents the residual error of the module, and the weight layer represents the weight of this layer. *X* is an input value, and *F* (*x*) is the output after linear change and activation of the first layer. Between the linear change in the second layer and activation, the input value of the current layer *X* is added, and then *F* (*x*) is output after activation.

### 2.3. Superresolution Reconstruction Algorithm of Mural Images to Enhance Artistry

**Figure 3:**
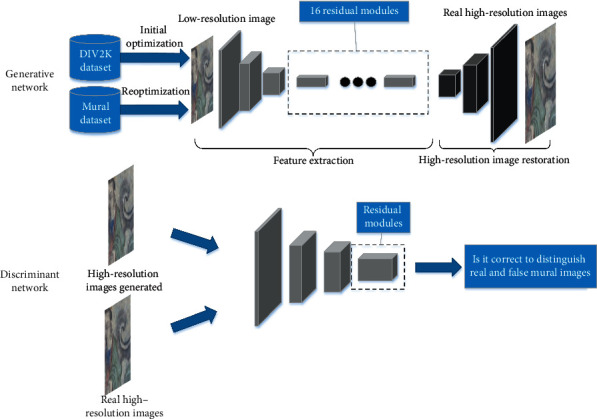
Structure of the proposed algorithm for the superresolution reconstruction of artistic mural images.

Based on the characteristics of ancient murals and image restoration algorithms, this study designs a new algorithm for the superresolution reconstruction of artistic mural images. The overall structure of the algorithm is shown in Figure 3, which mainly focuses on three aspects: the network structure design, loss function, and the training and testing process.

#### 2.3.1. Network Structure Design

The mural image optimization network is divided into two parts: the generative network and the discriminant network. The generative network aims to output high-resolution images after superresolution reconstruction. The discriminant network aims to determine the authenticity of the output image of the generative network and the real mural image.

The design architecture of the generative network follows the encoder-decoder structure, which is mainly divided into feature extraction and image reconstruction. The input of the network is a low-resolution mural image and the output is a high-resolution image corresponding to the input image.

In the feature extraction, 16 residual modules are introduced for feature extraction based on the idea of residual learning. It is worth noting that when dealing with high-level computer vision problems, such as image classification, batch normalization (BN) is usually integrated before each activation function of the convolution layer of the neural network to speed up the training time of the network model and solve the problems of gradient explosion and gradient dispersion [27].

**Figure 4:**
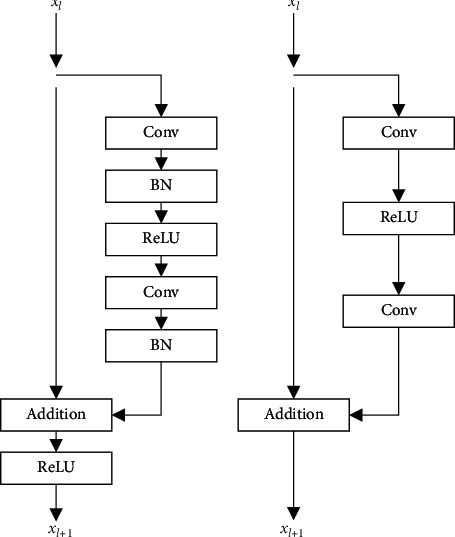
The comparison of residual modules. (a) Traditional residual module. (b) Improved residual module.

When dealing with the problem of low-level computer vision and the deep network trained under the GAN framework, the addition of the BN layer will produce artifacts, consume more computing performance, and reduce the effect of image superresolution reconstruction. In such a situation, the BN operation is removed from the residual module to further optimize the network structure. The comparison between the traditional residual module and the residual module used in this study is shown in Figure 4.

After the low-resolution features are extracted, superresolution image restoration is performed, followed by the final output of the high-resolution image. During upsampling, this study uses subpixel convolution instead of transposition convolution. Subpixel convolution uses a normal convolution structure, but the output channel is related to the target resolution. A shuffle operation is performed over the channel to obtain the output whose resolution is the same as that of the target. Compared with transposed convolution, the best feature of subpixel convolution is that the receptive field of the feature map is larger, which can provide more image information for superresolution reconstruction.

The main purpose of the discriminant network is to accurately classify real superresolution images and superresolution image output by the generative network. With the improvement in classification accuracy, it promotes the optimization of the generative network, thereby producing high-quality, high-resolution images.

The discriminant network consists of the input layer, convolution layer, and fully connected layer. Network inputs include authentic high-resolution images and generated high-resolution images. To extract higher-dimensional image features, 11 convolution layers are used. The 9–11th layers form the residual module, and the outputs of the 8th layer and 11th layer are then summed to obtain the final image features. This treatment makes the network avoid gradient dispersion and other problems to some extent. Finally, the discriminant network design is completed after the classification of the fully connected layer. The details of the discriminant network are summarized in Table 1.

#### 2.3.2. Loss Function

The loss function of the generative network consists of content loss (*l*_*X*_^SR^) and adversarial network loss (*l*_Gen_^SR^). The loss function of the generative network is calculated as follows:

(2) $$\begin{array}{c} l^{\text{SR}}=l_{X}^{\text{SR}}+10^{-3}l_{\text{Gen}}^{\text{SR}}. \end{array}$$

Content loss includes MSE loss (*l*_MSE_) and VGG loss (*l*_VGG_). Generally, mean square error loss is used for network optimization to obtain high-resolution images with high similarity at the pixel level. MSE is the sum of the square of the distance between the target variable and the predicted value of each sample. MSE is calculated, which is the sum of all squared losses for each sample, and then is divided by the number of samples. The MSE loss function is as follows:

(3) $$\begin{array}{c} l_{\text{MSE}}=\frac{1}{N}\sum_{\left(x,y\right)\in D}{\left(y-\text{prediction}\left(x\right)\right)}^{2}, \end{array}$$

where *N* refers to the number of samples, (*x*, *y*) refers to the sample (*x* is the feature set in the training sample, and *y* is the real value in the training sample), and prediction (*x*) refers to the predicted value of the sample.

The mere use of the MSE loss function is likely to produce local area smoothing, which creates difficulty in recovering lost high-frequency details, such as texture information. Therefore, the VGG loss function is integrated. The VGG loss function obtains the difference in the feature map between the real high-resolution image and the generated high-resolution image and then optimizes the model in a higher feature dimension using a gradient descent algorithm. Specifically, the generated high-resolution image and real high-resolution image are input into the pretrained 19-layer VGG network. Based on the feature map obtained after VGG network processing, the Euclidean distance is calculated, which is taken as the VGG loss. The calculation formula of VGG loss is as follows:

(4) $$\begin{array}{c} l_{\text{VGG}\left(i,j\right)}=\frac{1}{W_{i,j}H_{i,j}}\sum_{x=1}^{W_{i,j}}\sum_{y=1}^{H_{i,j}}{\left(\phi_{i,j}{\left(I^{\text{HR}}\right)}_{x,y}-\phi_{i,j}{\left(G_{\theta_{G}}\left(I^{\text{LR}}\right)\right)}_{x,y}\right)}^{2}, \end{array}$$

where *i* and *j* represent the *j*th convolution (after activation) before the *i*th pooling layer, *W* and *H* represent the width and height of the feature map, respectively, *I*^HR^ represents the real high-resolution image, *I*^LR^ represents the low-resolution image, *G*_*θ*_*G*__(*I*^LR^) is the superresolution image of the low-resolution image generated by the network model, and *ϕ*_*i*,*j*_(*I*^HR^)_*x*,*y*_ − *ϕ*_*i*,*j*_(*G*_*θ*_*G*__(*I*^LR^))_*x*,*y*_ is the difference between the real superresolution image and the generated superresolution image in the feature map obtained through the VGG19 network.

Finally, the idea of adversarial learning is introduced into the network, and the generative adversarial loss is included in the calculation of the loss function to further optimize the generative network model. The calculation formula of the generative adversarial loss is as follows:

(5) $$\begin{array}{c} l_{\text{Gen}}^{\text{SR}}=\sum_{n=1}^{N}-\log\;D_{\theta_{G}}\left(G_{\theta_{G}}\left(I^{\text{LR}}\right)\right), \end{array}$$

where *D*_*θ*_*G*__(*G*_*θ*_*G*__(*I*^LR^)) represents the probability that the high-resolution image generated by the generative network is identified as the real high-resolution image by the discriminant network.

#### 2.3.3. Training-Testing Flow Sheet

In this study, the training process is divided into the training of the generative network and that of the generative network combined with the discriminant network. The specific training algorithm for the network model is described as follows (Algorithm 1).

**Figure 5:**
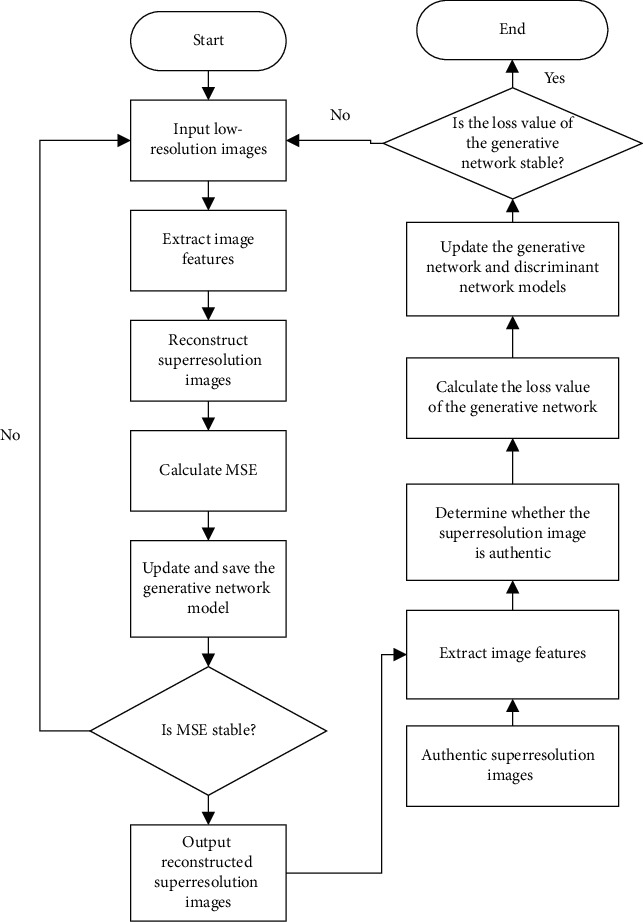
The training flow of the proposed model.

The flowchart of the model training is shown in Figure 5.

After model training, the generated network model and discriminant network model are finally obtained. Then, the generative network model is tested. In the testing process, the basic processes are basically consistent with those of the training processes, except that the parameters of the network model are no longer updated.

## 3. Results and Discussion

### 3.1. Experimental Design

Dataset: In this study, the public-open DIV2K dataset combined with a small number of mural image datasets is used to complete the construction of the network model. The DIV2K contains 800 pairs of images with various types and rich features, and the mural datasets contain 100 pairs of ancient high-quality mural images, which, to a certain extent, solves the problem of the adaptation of the depth domain. In the process of training, we use DIV2K and 50 pairs of mural images to complete the construction of the model. In the test process, 50 other pairs of mural images are used to collect the results data analysis. The verification dataset in this study is ancient mural images. The comparative experiment is divided into objective index comparison and subjective evaluation comparison to make the experiment more complete and the experimental results more convincing.

Experimental environment: The effectiveness of the proposed algorithm is verified. The hardware environment primarily consists of an Intel core i5-9400fF@2.90 GHz, with 16 GB of memory and a Nvidia GeForce RTX2070 video card. The software environment is Python 3.7 for language programming on the Windows 10 system, with TensorFlow as the framework to complete the superresolution reconstruction of mural images.

### 3.2. Experimental Results and Analysis

**Figure 6:**
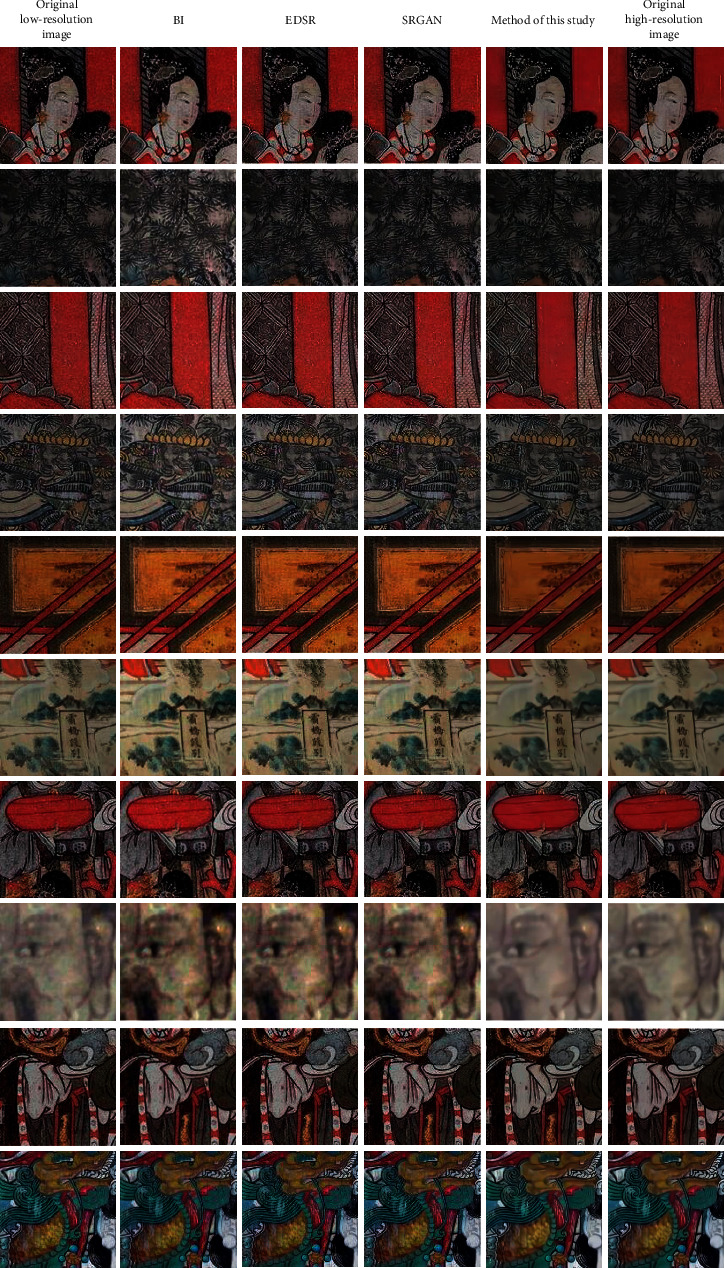
Comparison of superresolution reconstruction effects of different mural images under different algorithms.

Ten mural images of different styles with different color contrast and rich texture details were locally magnified four times, and the superresolution reconstruction effect of the proposed algorithm was compared with those of the bicubic interpolation (BI) algorithm [6], EDSR algorithm [19], and SRGAN algorithm [20]. The results are shown in Figure 6.

As shown in Figure 6, the superresolution images restored by the interpolation-based BI algorithm appear blurred with zigzagged image texture. This is because this algorithm assumes that the gray value of image pixels changes continuously and smoothly. However, this assumption is not in line with the actual situation. Additionally, this algorithm does not consider the degradation model of the image, resulting in unsatisfactory superresolution. Currently, the deep-learning-based EDSR algorithm and SRGAN algorithm are extensively used in practice. Compared with the BI algorithm, these two greatly improved the repair effect.

However, due to the small number of network layers used in these algorithms for image feature extraction, more image details cannot be obtained. This drawback results in a blurred superresolution reconstruction effect at the edge region of the image. Moreover, large deviations may sometimes exist in the optimization of image color based on these algorithms, and, therefore, the restoration effect on reconstructed image details needs to be improved. Compared with the above-mentioned superresolution reconstruction algorithms, the algorithm proposed in this study achieves a better effect on the superresolution reconstruction in terms of texture information and color saturation.

#### 3.2.1. Subjective Assessment

Comparisons in objective indices may not fully reflect the human visual perception of the mural superresolution reconstruction image. To make the superresolution image reconstruction more universal, we also selected five experts in the field of mural work and 20 professionals with normal vision to score the optimization effect of the four different algorithms. The highest score was 10, and the lowest was 1. The quality of the mural image was judged according to the score.

**Figure 7:**
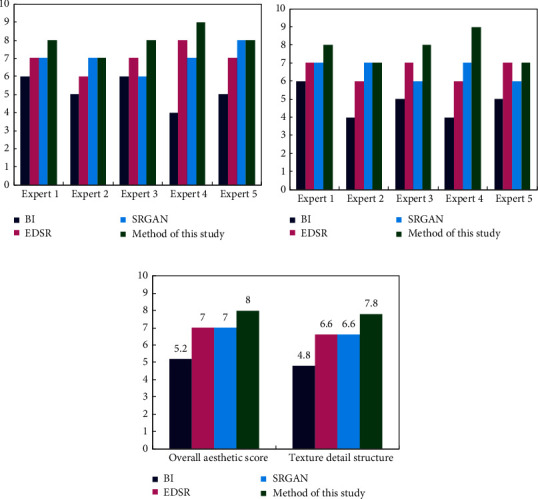
The score of five experts under the different algorithms. (a) The score of the overall esthetics. (b) The score of the detailed texture structure. (c) The average score.

All the selected experts performed much research work in the field of murals and have a deep understanding of murals. The selection of experts for scoring enables the comparative results to be more referential and authoritative. Ten representative mural images were selected by the five experts, and the optimization effects of the four algorithms were evaluated from the perspectives of overall esthetics and texture details. The scores assigned by the experts in terms of overall esthetics are shown in Figure 7(a), which reflects whether the overall color of the reconstructed images is rich and in line with the artistic conception of the mural. The scores assigned by the experts in terms of texture structure are shown in Figure 7(b), which reflects whether the changes in the lines and texture color are consistent with the painting habits of the murals. The average scores are shown in Figure 7(c), which can avoid scoring contingency and more scientifically exhibit the advantages and disadvantages of different algorithms.

The purpose of mural image optimization is not only to protect ancient cultural relics but also to encourage ordinary people to learn and appreciate the beauty of ancient murals. For this reason, we selected 20 people from different work positions to score 5 mural images optimized by different algorithms. The average scores of each algorithm were obtained. These scores represent the recognition of the vast majority of people for different quality mural images and the excellence of the corresponding algorithm. Therefore, the score results were more universal. The scoring results are shown in Table 2.

Compared with other superresolution reconstruction algorithms, the experts in the field of murals gave a higher evaluation of the algorithm proposed in this study in terms of overall esthetics and texture detail structure, which reflects the effectiveness and superiority of the algorithm in the professional field. Similarly, the volunteers from different industries also gave a higher score for the mural image optimized under the proposed algorithm, which shows that the mural images optimized by the algorithm in this study achieved satisfactory results. Therefore, the algorithm proposed in this study also outperformed other algorithms in terms of subjective evaluation.

#### 3.2.2. Objective Assessment

In addition to the three algorithms mentioned above, this study also selects four recently proposed algorithms that are representative in the field of the superresolution reconstruction of images, and the algorithm obtained after the discriminant network is removed from the algorithm proposed in this study, for comparisons in terms of PSNR, SSIM, natural image quality evaluator (NIQE), and inference time.

PSRN evaluates the quality of the image by comparing the differences between the corresponding pixels of the two images. A higher PSNR indicates a smaller distortion and better superresolution reconstruction effect. The PSNR is calculated as follows:

(6) $$\begin{array}{c} \text{PSNR}=10\;\log_{10}\frac{255^{2}\times W\times H}{\sum_{i=1}^{W}\sum_{j=1}^{H}{\left[X\left(i,j\right)-Y\left(i,j\right)\right]}^{2}}, \end{array}$$

where *W* is the width of the image, *H* is the height of the image, and *X* (*i, j*) and *Y* (*i, j*) represent the pixel values of two superresolution images.

SSIM is an index for evaluating image similarities in terms of brightness, contrast, and structure. The value range of SSIM is [0, 1], and a higher value indicates higher similarity. SSIM is calculated as follows:

(7) $$\begin{array}{c} \text{SSIM}\left(x,y\right)=\frac{\left(2\mu_{x}\mu_{y}+c_{1}\right)\left(2\sigma_{xy}+c_{2}\right)}{\left(\mu_{x}^{2}+\mu_{y}^{2}+c_{1}\right)\left(\sigma_{x}^{2}+\sigma_{y}^{2}+c_{2}\right)}, \end{array}$$

where *x* and *y* represent the reconstructed superresolution image and the original high-resolution image, respectively; *μ*_*x*_ and *μ*_*y*_ are the average values of *x* and *y*, respectively; *σ*_*x*_^2^ and *σ*_*y*_^2^ are the variances in *x* and *y*, respectively; *σ*_*xy*_ is the covariance of *x* and *y*; and *c*_1_ and *c*_2_ are constants.

NIQE serves as an objective evaluation index, which extracts features from natural landscapes for image testing [28]. The extracted features are fitted into a multiple Gaussian model, which is responsible for measuring the difference in the multivariate distribution of the image to be tested (the distribution is constructed with the features extracted from a series of normal natural images).

The experimental results of various algorithms in terms of PSNR, SSIM, NIQE, and inference time are summarized in Table 3.

As shown in Table 3, the performance of the algorithm proposed in this study ranks the first in SSIM and the second in PSNR and NIQE. However, the proposed algorithm shows poor performance in terms of inference time, which ranks sixth among all considered algorithms. This is because the algorithm proposed in this study uses a more complex network structure for image feature learning, which obtains better quality of images at cost of the inference speed.

Compared with the classic algorithms BI, EDSR, and SRGAN, the proposed algorithm increases PSNR by 1.2–3.3 dB and SSIM by 0.04–0.13. Compared with the algorithms used in the latest research in the field of image superresolution reconstruction, that is, literature [21], literature [22], literature [23], and literature [24], the algorithm proposed in this study also exhibits overall more satisfactory experimental results: It reduces the algorithm's interference time while obtaining higher image quality. Compared with the algorithm used in the ablation study, the proposed algorithm in this study has greatly improved image quality, which illustrates the effectiveness of the algorithm improvement in this study, although the improvement increases the inference time.

Based on the above analyses, the algorithm proposed in this paper fully exhibits its effectiveness and excellence in improving the quality of mural images.

## 4. Conclusions

Aiming at mural image optimization, this study proposed a superresolution reconstruction algorithm to enhance the artistry of mural images.

A CNN is used as the infrastructure to realize feature extraction for the mural images. The generative network is optimized by residual learning, and then the superresolution reconstruction of the mural image is realized based on the extracted features through the upsampling of the subpixel convolution. In the discriminant network, the deep convolutional neural network and residual modules are used to distinguish between the generated high-resolution images and the real high-resolution images. Different from common single loss functions, the algorithm proposed in this study adopts a combination of multiple loss functions. Additionally, it uses the method of staged network model optimization to realize the scientific transformation process from low-resolution images to high-resolution images. Compared with existing algorithms, mural images optimized by the proposed algorithm have noticeable improvement according to the subjective visual effect and objective experimental data. The results show that this algorithm has a better effect on the superresolution reconstruction of mural images with rich color and strong texture structure.

However, this algorithm also suffers from some drawbacks. In superresolution reconstruction of mural images, noise of other colors often appears in regions with a single color and strong contrast, which makes the image color impure and reduces the artistic value. The training time of the adversarial neural network is uncertain. In the future, we will fuse superresolution reconstruction with an image noise reduction algorithm to solve the phenomenon of noise in high-resolution images. We will also conduct research by adopting more scientific GAN training termination conditions to realize superresolution reconstruction of mural images.

## Figures and Tables

**Algorithm 1:**
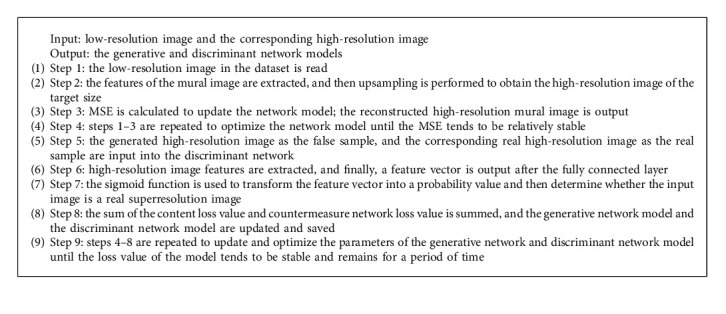
Algorithm training process.

**Table 1:** Details of the discriminant network.

Name	Type	Kernel	Stride	Outputs
Conv1	Conv	4 × 4	2 × 2	64
Conv2	Conv	4 × 4	2 × 2	128
Conv3	Conv	4 × 4	2 × 2	256
Conv4	Conv	4 × 4	2 × 2	512
Conv5	Conv	4 × 4	2 × 2	1024
Conv6	Conv	4 × 4	2 × 2	2048
Conv7	Conv	1 × 1	1 × 1	1024
Conv8	Conv	1 × 1	1 × 1	512
Conv9	Conv	1 × 1	1 × 1	128
Conv10	Conv	3 × 3	1 × 1	128
Conv11	Conv	3 × 3	1 × 1	512
FC	FC	—	—	1

**Table 2:** The score of different industry staff.

Algorithm	Personnel																				Ave
	1	2	3	4	5	6	7	8	9	10	11	12	13	14	15	16	17	18	19	20	
BI	5	5	5	6	5	5	6	7	7	6	5	5	4	6	5	5	7	5	4	5	5.45
	6	4	6	5	6	5	4	6	6	6	6	6	5	5	6	5	6	6	6	6	
	6	6	5	4	5	5	5	8	4	7	7	6	5	5	4	5	5	6	5	7	
	4	5	4	4	4	6	6	6	5	8	4	7	6	5	5	6	5	5	6	5	
	4	5	6	6	6	6	7	4	7	8	5	4	5	4	6	7	5	4	6	5	

EDSR	5	6	6	5	6	6	5	5	6	6	7	6	6	6	7	7	6	7	6	6	6.44
	6	7	7	6	8	7	6	7	5	7	6	7	6	7	7	5	6	8	7	7	
	6	6	7	7	7	6	7	6	7	8	6	6	5	6	7	6	8	7	6	6	
	7	5	7	6	5	5	8	7	8	8	5	7	7	8	6	6	7	7	6	6	
	7	7	6	5	6	6	6	8	6	8	7	7	6	7	7	7	5	5	8	7	

SRGAN	6	6	6	7	7	6	5	6	7	7	4	7	7	6	6	6	6	6	7	8	6.55
	5	7	7	8	6	7	7	7	8	8	6	7	6	8	7	6	7	6	8	7	
	6	6	8	6	8	6	6	8	8	7	7	8	5	5	8	6	6	7	6	8	
	7	6	8	5	7	5	6	6	7	7	8	5	7	6	7	8	5	5	7	6	
	8	7	6	5	6	5	7	6	7	7	6	6	6	8	7	7	6	5	8	5	

Method of this study	7	8	7	8	6	7	8	7	8	8	7	8	7	8	7	7	8	8	8	8	7.44
	7	8	8	7	7	8	7	9	7	7	7	8	6	9	8	7	9	8	9	7	
	6	8	8	7	8	6	6	6	8	9	8	6	8	6	8	8	8	6	8	8	
	8	7	8	6	7	5	6	7	7	8	9	7	6	8	7	8	7	7	8	9	
	9	7	7	8	6	8	8	8	9	8	6	8	6	7	8	7	8	8	7	7	

**Table 3:** Experimental results of various algorithms.

Algorithm	PSNR	SSIM	NIQE	Inference time (ms)
BI	24.96	0.687	0.883	75
EDSR	27.12	0.768	0.912	103
SRGAN	27.01	0.771	0.923	246
Literature [21]	27.51	0.763	0.951	305
Literature [22]	28.13	0.781	0.946	326
Literature [23]	27.92	0.792	0.975	363
Literature [24]	28.63	0.803	0.963	471
Ablation study	25.85	0.713	0.881	204
Method of this study	28.36	0.813	0.967	322

## Data Availability

All data for the analysis in this study are included within the article.
